# Sexually dimorphic peripheral sensory neurons regulate copulation duration and persistence in male *Drosophila*

**DOI:** 10.1038/s41598-022-10247-3

**Published:** 2022-04-13

**Authors:** Shreyas Jois, Yick-Bun Chan, Maria Paz Fernandez, Narsimha Pujari, Lea Joline Janz, Sarah Parker, Adelaine Kwun-Wai Leung

**Affiliations:** 1 Department of Veterinary Biomedical Sciences, WCVM, University of Saskatchewan, Saskatoon, SK S7N 5B4 Canada; 2 Department of Neurobiology, Harvard Medical School, Boston, MA 02115 USA; 3 Department of Neuroscience and Behavior, Barnard College, New York City, NY 10027 USA; 4 Centre for Applied Epidemiology, Large Animal Clinical Science, WCVM, University of Saskatchewan, Saskatoon, SK S7N 5B4 Canada

**Keywords:** Neural circuits, Sexual dimorphism

## Abstract

Peripheral sensory neurons are the gateway to the environment across species. In *Drosophila*, olfactory and gustatory senses are required to initiate courtship, as well as for the escalation of courtship patterns that lead to copulation. To be successful, copulation must last long enough to ensure the transfer of sperm and seminal fluid that ultimately leads to fertilization. The peripheral sensory information required to regulate copulation duration is unclear. Here, we employed genetic manipulations that allow driving gene expression in the male genitalia as a tool to uncover the role of these genitalia specific neurons in copulation. The fly genitalia contain sex-specific bristle hairs innervated by mechanosensory neurons. To date, the role of the sensory information collected by these peripheral neurons in male copulatory behavior is unknown. We confirmed that these MSNs are cholinergic and co-express both *fru* and *dsx*. We found that the sensory information received by the peripheral sensory neurons from the front legs (GRNs) and mechanosensory neurons (MSNs) at the male genitalia contribute to the regulation of copulation duration. Moreover, our results show that their function is required for copulation persistence, which ensures copulation is undisrupted in the presence of environmental stress before sperm transfer is complete.

## Introduction

An animal’s decision to perform a certain behavior requires the integration of external stimuli with homeostatic regulation. The nervous system collects environmental sensory information and integrates it with the internal status of the animal to generate motor signals that elicit an appropriate behavioral response. *Drosophila* male courtship is an excellent model to investigate how complex behavior is coordinated by the nervous system. The development of the courtship neural circuit is largely controlled by two sex determination genes, *fruitless (fru)* and *doublesex (dsx)*^1–3^*.* The ~ 2000 *fru*-expressing neurons, comprising of sensory, integration, and motor neurons, control most aspects of male courtship behavior^4–9^. However, the complete courtship neural circuit also requires *dsx-*expressing neurons, a subset of which co-expresses *fru*^10–14^.

Successful courtship requires the processing of sensory information received by peripheral sensory neurons^15^. In the antenna, *fru* olfactory receptor neurons (ORNs) receive and transmit volatile olfactory signals to the antennal lobe in the brain; *fru* ORNs expressing the receptor Or67d respond to the male pheromone cVA which inhibits mating behavior in males but promotes mating behaviors in females^16^. Gustatory receptor neurons (GRNs) in the front legs receive and transmit non-volatile gustatory signals to the CNS. For example, *fru* GRNs expressing the ion channels ppk23 and ppk29 are required to detect inhibitory signals on males and excitatory signals on females during courtship^17^. *Dsx* neurons expressing the receptor Gr68a respond to the anti-aphrodisiac pheromone CH503 and inhibit male courtship^18^. Acoustic information from the movement of the female detected by the Johnston’s organ neurons (JONs) helps to promote courtship initiation in males^19^. Finally, the visual contribution to the initiation and maintenance of courtship has also been shown in a treadmill-based courtship assay^20^.

When the female is receptive to male courtship, she will slow down and spread her wings, allowing the male to mount. The male initiates copulation by bending his abdomen forward to attach the genitalia. The male terminalia is divided in two regions: the periphallic structures and the intromittent organ. The periphallic structures form weaker connections to the intromittent organ and can be easily separated by dissection. The periphalilic structures are subdivided into four regions: the epandrial ventral lobe (formerly called: lateral plate) (EVL), the surstylus (formerly called: clasper) (SUR), the epandrial posterior lobe (formerly called posterior lobe) (EPL), and the cercus (formerly called: anal plate) (CER)^21^ (Fig. 2A). Genital coupling, as revealed by a high-resolution electron microscopic time sequence analysis, involves the active movement of these periphalic structures^22^. The surstylus bends medially and is hidden from view. Within 10 min of copulation, the cercus aligns with the female oviscape and achieves genital coupling. Each periphallic region contains an array of stereotypic, species and sex-specific bristles, and each bristle is innervated by a bipolar mechanosensory neuron^23^. The neural implication of this sensory information collected during copulation is unclear.

Copulation must last long enough to ensure successful sperm transfer, which does not occur until five minutes after copulation starts^24,25^. The regulation of copulation time in *Drosophila* is a complicated process that does not simply depend on the volume or the transfer of sperm and seminal fluid, since mutants defective in their synthesis still have normal copulation duration^24^. Both neuronal and non-neuronal factors can influence mating time. Neuronal regulators include a small cohort of *fru* neurons that co-express the transcription factor *engrail*^26^ and a subset of 4–5 *fru* neurons that innervate the male reproductive tissues^27,28^. The latter neurons control copulation duration in response to sperm transfer^27^. Non-neuronal factors include mutations in the circadian clock gene *period*, which exhibit longer copulation duration^29^, and environmental factors such as the gut microbiome^30,31^, the social environment of the male^32–35^, and environmental stressors^24^. Copulation duration reflects the reproductive investment of the males^32^. Sustaining copulation with a suboptimal mate is a loss of opportunity to gain a better mate. Therefore, males must respond to extrinsic and intrinsic factors by adjusting copulation duration to maximize reproductive success^33^. During the first 5 min before sperm transfer occurs, the benefit of maintaining copulation outweighs any risk because terminating copulation will lead to no fertilization. Copulation persistence describes the maintenance of copulation in the presence of stressful stimuli, which peaks within the first 5 min of copulation and decreases over time^24^. This is an important cost–benefit analysis that ensures species survival. The neural circuit for this cost–benefit analysis includes 8 *dsx*/GABA neurons in the ventral cord that decrease copulation persistence. With opposite action, ventral cord DA neurons increase copulation persistence^24^. However, the neural input required to initiate copulation persistence is currently unknown. In this study, we show that sensory information received by the peripheral sensory neurons from the front legs (GRNs) and mechanosensory neurons (MSNs) at the male genitalia contribute to the regulation of copulation duration and their function is critical for the maintenance of copulation in the presence of stressful stimuli before sperm transfer is complete.

## Results

### Discovery of *fruitless (fru)* neurons that regulate copulation duration

**Figure 1:**
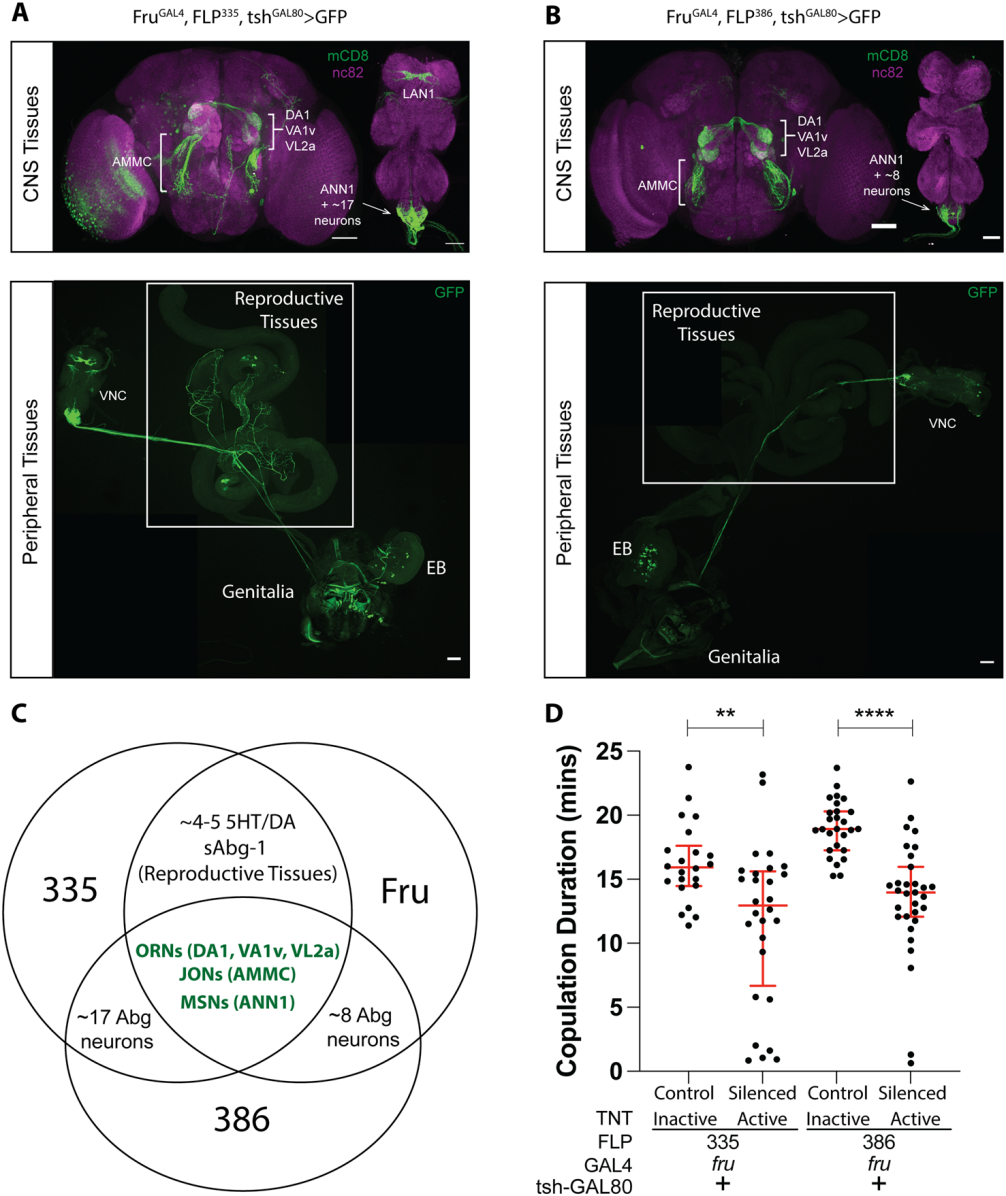
Different groups of *fru* neurons regulate copulation duration. (**A**, **B**) Comparison of *fru* neuronal expression restricted by two different FLP lines. Expression profile of Fru^GAL4^, FLP^335^, tsh^GAL80^ > mCD8::GFP (**A**) is compared to that of Fru^GAL4^, FLP^386^, tsh^GAL80^ > mCD8::GFP (**B**). CNS tissues were stained with anti-mCD8 (green) and nc82 (magenta) and peripheral tissues were stained with anti-GFP (green) only. Expression in the reproductive tissues was notably eliminated via FLP^386^. Scale Bar = 50 µm. (Video 1) Confocal stack of the male genitalia of Fru^GAL4^, FLP^386^, tsh^GAL80^ > mCD8::GFP showing individual neurons at the clasper. Tissue was stained with anti-GFP. (**C**) Venn diagrams highlighting the common *fru* neurons targeted by both FLP lines as shown in (**A**) and (**B**). Names of the arbors are in brackets followed by the names of the neuronal cluster where they are originated. Three common *fru* neurons were consistently labeled by both FLP lines: (1) the sexually dimorphic projections in the glomeruli from *fru* ORNs (DA1, VA1v, VL2a), (2) projections in the antennal mechanosensory motor complex (AMMC) from *fru* JONs, (3) projections in the abdominal ganglion (ANN1) from *fru* MSNs in the genitalia. See also Table 1 in text. (**D**) Effect of silencing FLP^335^ and FLP^386^ restricted labeling of *fru*-GAL4. A shortened copulation duration was observed in both genetic combinations, therefore, the common neuronal clusters targeted by both FLP lines (ORNs, JONs, MSNs) were responsible for the copulation phenotype. Black dots are individual values. Red lines indicate the median with interquartile range (n = 22–31). **p < 0.0034, ****p < 0.0001 by Mann Whitney test.

To uncover the neuronal regulators of copulation duration, we utilized an intersectional genetic approach, involving both the GAL4/UAS^36^ and FLP/FRT^7^ expression systems. The details of the intersectional genetic system and how we generated a FLP enhancer trap screen had been described elsewhere^28,37,38^. Briefly, the target gene is downstream of a DNA sequence that contains the binding site (UAS) for the transcription factor GAL4, followed by a DNA sequence that contains a stop codon flanked by the FLP recombinase recognition site FRT (UAS-FRT-stop-FRT-target gene). A cell must possess an active promoter for FLP (to remove the stop codon by recombination) and an active promoter for GAL4 (to bind to UAS) to activate expression of the target gene. As an additional tool to restrict gene expression, tsh^GAL80^ is used to inhibit GAL4 expression specifically in the ventral cord. From here forward, the genetic nomenclature will list all transgenes that are responsible to drive expression of the target gene (e.g. “X^GAL4^,FLP^#^ > target gene” denotes a fly that carries a GAL4 driver controlled by the promoter of X, and the FLP line # from the enhancer trap screen, expressing the target gene). For the relevant neuronal populations, we expressed either a membrane tagged GFP (UAS-FRT-stop-FRT-mCD8::GFP) to visualize the arborizations of the neurons or the neuronal silencer tetanus toxin (UAS-FRT-stop-FRT-TNT) to block neuronal activity. Previously, we identified a cluster of ~ 5 *fru* 5HT/DA neurons (*fru-*sAbg-1) in the male abdominal ganglion that innervate various male reproductive tissues and regulates copulation duration^28^. In that study, we observed a reduction in copulation duration when we combined FLP335 with either *fru-*GAL, that targets most fruitless neurons (Fru^GAL4^, FLP^335^, tsh^GAL80^ > TNT), with Th-GAL4, that targets dopaminergic neurons (Th^GAL4^, FLP^335^, tsh^GAL80^ > TNT), or with 5HT-GAL4, that targets serotonergic neurons (5HT^GAL4^, FLP^335^, tsh^GAL80^ > TNT)^28^. However, neither silencing the subsets of dopaminergic nor serotonergic neurons can recapitulate the copulation phenotype observed when we targeted the broader fruitless circuit. The result suggested that additional *fru* neurons contributed to the copulation duration phenotype^28^. Indeed, the expression pattern for Fru^Gal4^, FLP^335^, tsh^GAL80^ > mCD8::GFP males extended beyond the accessory glands and seminal vesicles all the way to the genitalia (Fig. 1A). Upon further examination, we were able to trace GFP expression to the male genitalia.

To further confirm the genitalia neurons are implicated in copulation duration control, we screened our enhancer trap FLP library^28^ to search for other FLP lines that affected copulatory behaviors. We identified another FLP line that showed expression at the genitalia and also reduced copulation duration when it is combined with *fru-*GAL4 to express TNT in males (Fru^GAL4^, FLP^386^, tsh^GAL80^ > TNT) (control:Inactive TNT/silenced:Active TNT) (Fig. 1D). The experimental males (Fru^GAL4^, FLP^386^, tsh^GAL80^ > mCD8::GFP) showed similar expression in *fru* ORNs, JONs, and GRNs compared to Fru^GAL4^, FLP^335^, tsh^GAL80^ > mCD8::GFP males (Fig. 1B). However, the prominent *fru*-sAbg-1 neurons in the abdominal ganglion that innervate the male reproductive tissue were noticeably missing, leaving only the sexually dimorphic arborizations (ANN1) in the abdominal ganglion originating from the MSNs in the genitalia. The expression pattern in the peripheral tissues that extend out from the ventral cord of Fru^GAL4^,FLP^386^,tsh^GAL80^ > mCD8::GFP males was highly restricted, showing GFP expression only in the male genitalia (Fig. 1B, video of confocal stacks). Although we still observed expression for the *fru* LAN1 arborations originating from the GRNs, it is less consistent compared to Fru^GAL4^,FLP^335^, tsh^GAL80^ > mCD8::GFP. In summary, three common *fru* neurons are consistently labeled by both FLP lines: (1) the sexually dimorphic projections in the glomeruli from *fru* ORNs (DA1, VA1v, VL2a), (2) projections in the antennal mechanosensory motor complex (AMMC) from *fru* JONs, and (3) projections in the abdominal ganglion (ANN1) from *fru* MSNs in the genitalia (Fig. 1C, Table 1). The copulation duration of Fru^GAL4^, FLP^386^, tsh^GAL80^ > TNT was shortened by ~ 26% compared to the control (Fig. 1D). This difference is comparable to that observed in Fru^GAL4^, FLP^335^, tsh^GAL80^ > TNT males. Of the three groups of *fru* neurons targeted by both FLP lines, we hypothesized that ANN1 from *fru* MSNs in the genitalia were the most likely to contribute to the copulation duration phenotype since we showed previously that silencing *fru* ORNs and GRNs did not affect copulation duration^28^. Recently, it was discovered that females sing *in copula* and that this song influences the reproductive success of the male^39^. However, the study showed that copulation duration was unaffected by the absence of female singing^39^.

**Table 1:** Summary of *fru* expression restricted by FLP^335^ or FLP^386^ in combination with *fru*-GAL4 and tsh-GAL80.

Neurons	Arbors	FLP335	FLP386
ORNs (peripheral)	DA1 (brain)	+	+
	VA1v (brain)	+	+
	VL2a (brain)	+	+
JONs (peripheral)	AMMC (brain)	+	+
MSNs (peripheral)	ANN1 (VNC)	+	+
GRNs (peripheral)	LAN1 (VNC)	+	+
~ 5 5HT/DA Abg-1 (VNC)	Reproductive tissues (peripheral)	+	
~ 12 AbgNs uncharacterized (VNC)		+	
~ 8 AbgNs uncharacterized (VNC)			+

In brackets is the anatomical location of the neurons or arbors. *ORN* olfactory receptor neuron, *JON* Johnston’s organ neuron, *MSN* mechanosensory neuron, *GRN* gustatory receptor neuron, *AbgN* abdominal neuron, *5HT* serotonergic neuron, *DA* dopaminergic neuron, *VNC* ventral nerve cord.

### Characterization of *fru* genitalia mechanosensory neurons (MSNs)

**Figure 2:**
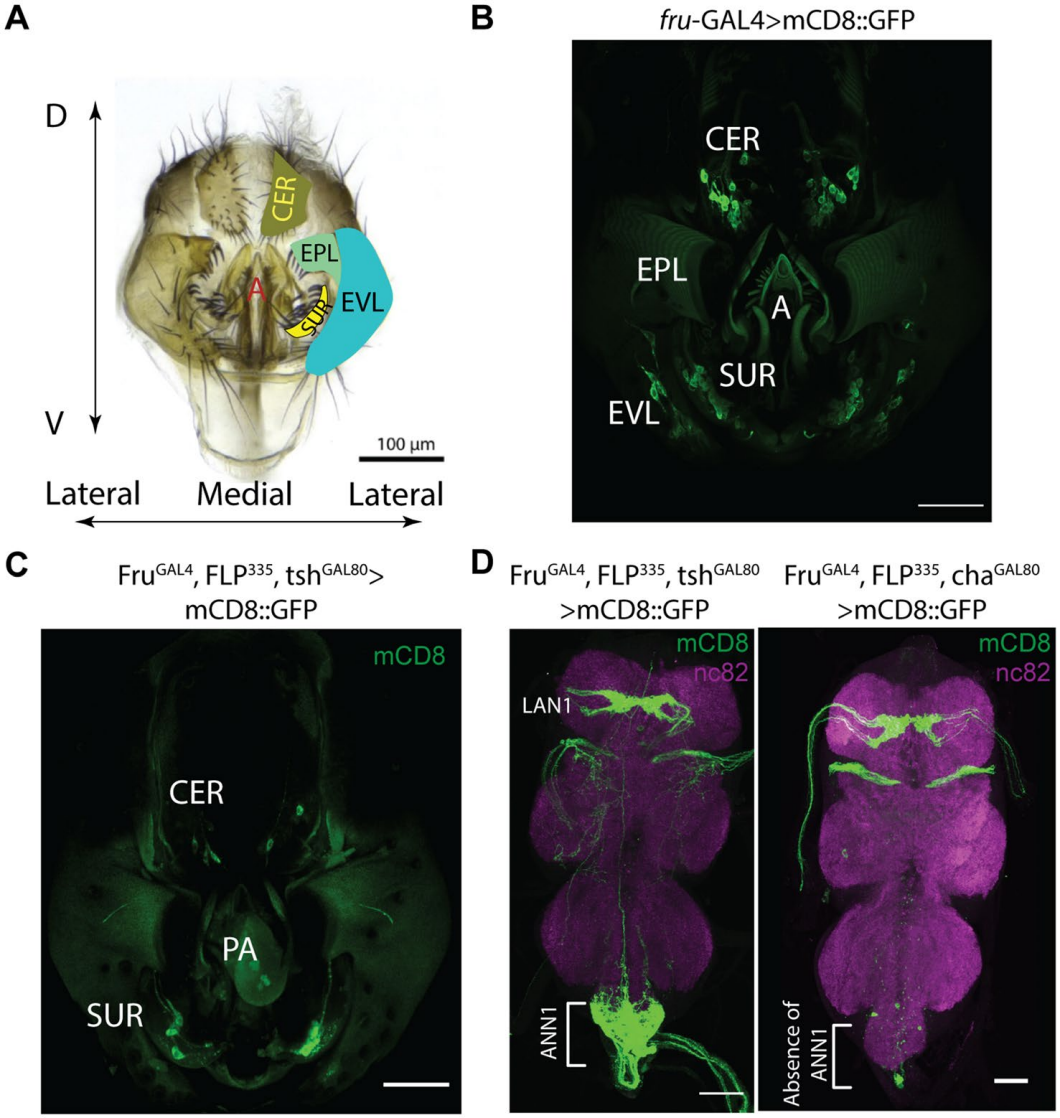
Characterization of *fru* neurons in the male genitalia. (**A**) Light microscope image of a male genitalia adapted from a previous work^21^. The male genitalia are comprised of the periphallic structures: cercus (CER), surstylus (SUR), epandrial ventral lobe (EVL) and the phallic structures: aedeagus (**A**). The phallic structures are attached to copulatory muscles (Fig. 5) and located just anterior to the periphallic structures. Stereotypical bristles on the surface of these cuticular plates are innervated by mechanosensory neurons (MSNs)^23^. (**B**) fru MSNs at the male genitalia. Expression profile of fru-GAL4 driving UASmCD8::GFP in the male genitalia. Fru expression was observed in ~ 50% of MSNs at the epandrial ventral lobe, ~ 64% of MSNs at the surstylus, and ~ 61% of MSNs at the cercus (Table 2). (**C**) fru MSNs restricted by FLP335. Expression profile of fru-GAL4, UAS > stop > mCD8::GFP, FLP335, tsh-GAL80 in the male genitalia. Adding FLP335 eliminated mCD8::GFP expression in the epandrial ventral lobe and restricted expression at the surstylus and the cercus (Table 2). The autofluorescence and 488 signals were unmixed as described in “Materials and methods”. (**D**) fru MSNs are cholinergic. Expression profile of FruGAL4,FLP335,ChaGAL80 > mCD8::GFP in the male ventral nerve cord. Adding the transgene cha-Gal80, which inhibits GAL4 in cholinergic neurons, eliminated ANN1 expression that originated from the genitalia MSNs (compare to Fig. 1A,B). Only fru-Abg-1 and other neurons in the abdominal ganglion showed expression. Scale Bar = 50 µm.

We further characterized the expression pattern in the genitalia terminals using various genetic combinations. The complete *fru* circuit (fru > mCD8::GFP) in males show consistent GFP expression in a subset of mechanosensory neurons in the epandrial ventral lobe (10 ± 4, ~ 50% of EVL bristles), the surstylus (16 ± 2, ~ 64% of SUR bristles), and the cercus (20 ± 2, ~ 61% of CER bristles) (Fig. 2A,B, Table 2). Adding FLP^335^ or FLP^386^ eliminated GFP expression in the epandrial ventral lobe and restricted expression in *fru* MSNs at the surstylus and the cercus (Fig. 2C, Table 2). Amongst these two lines, Fru^GAL4^, FLP^386^, tsh^GAL80^ > mCD8::GFP revealed a more restrictive expression pattern with 8 ± 3 *fru* neurons at the surstylus and 4 ± 2 at the cercus (Table 2). As Fru^GAL4^, FLP^386^, tsh^GAL80^ > TNT males resulted in the same copulation duration phenotype, we hypothesized that these 12 neurons (*fru*-MSNs, Table 2) at the genitalia are responsible for the shortened copulation duration phenotype. Since most sensory neurons in *Drosophila* are cholinergic, we hypothesized that *fru-*MSNs are the same. Indeed, using Cha-GAL80 that expresses the GAL4 inhibitor in cholinergic neurons eliminated ANN1 expression that originates from the genitalia MSNs (Fig. 2D).

**Table 2:** Expression summary of the male genitalia of the different genetic combinations.

Transgene present			Epandrial ventral lobe (EVL)		Surstylus (SUR)		Cercus (CER)		n
FLP	*fru-*Gal4	Tsh-Gal80	Percent sample showing expression (%)	Average number of cells	Percent sample showing expression (%)	Average number of cells	Percent sample showing expression (%)	Average number of cells	
−	−	−	−	20* ± 0.34		25* ± 0.63		32.7* ± 1.56	−
−	+	−	100	10 ± 4	100	16 ± 2	100	20 ± 2	12
335	+	+	0	−	100	10 ± 3	100	7 ± 2	10
386	+	+	0	−	94	8 ± 3	88	4 ± 2	16

*Based on bristle count published in Taylor 1998.

### *Fru* MSN neurons co-express *doublesex (dsx)*

**Figure 3:**
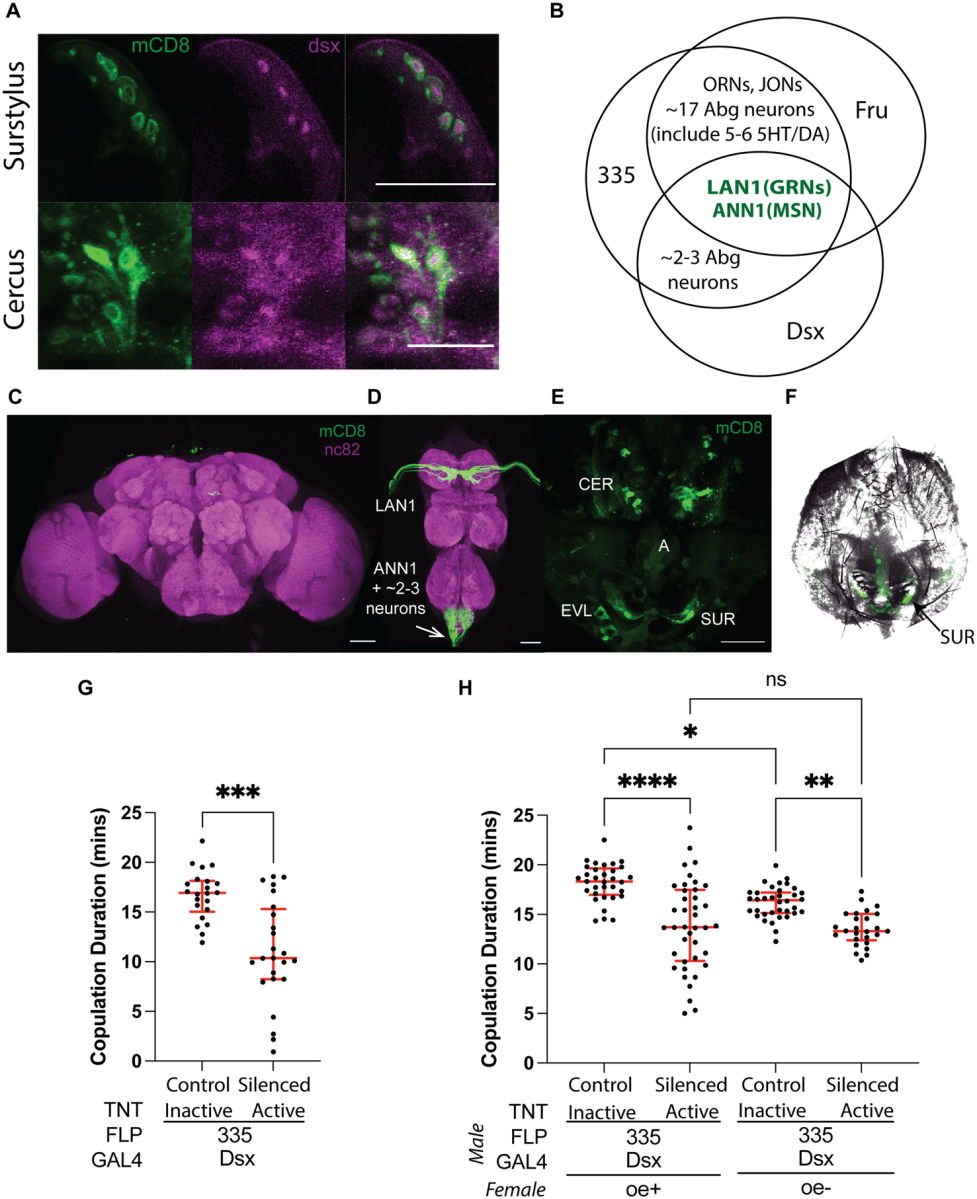
*ƒru*/*dsx* MSNs regulates copulation duration. (**A**) *fru* MSNs co-express *dsx*. Expression profile of Fru^GAL4^,FLP^335^ > FLP in the male genitalia. Double staining with anti-mCD8 (green) and anti-dsx (magenta) showed coexpression of *dsx* in *fru*-MSNs at the surstylus and the cercus of the genitalia. Scale Bar = 50 µm. (**B**) Venn diagram summarizing *fru* and *dsx* expression pattern restricted by FLP^335^. The sexually dimorphic LAN1 and ANN1 arbors originated from the GRNs on the front legs and MSNs of the genitalia were the only common expression observed in both GAL4 lines. (**C**–**F**) Expression profile of Dsx^GAL4^, FLP^335^ > mCD8::GFP. The CNS tissues were stained with anti-mCD8 (green) and anti-nc82 (magenta). No expression was observed in the brain (**C**), and the ventral nerve cord showed expression for the sexually dimorphic LAN1 arbors that originated from the GRNs in the front legs and the sexually dimorphic ANN1 arbors that originated from the MSNs in the genitalia (**D**). (**E**) The genitalia were stained with anti-mCD8 (green), autofluorescence (magenta). Expression was observed in the MSNs located on CER, SUR, and EVL of the genitalia. Scale Bar = 50 µm. (**F**) GFP expression on the genitalia overlaid with a DIC image of the cuticle. (**G**) Effect of silencing FLP^335^ restricted labeling of *dsx*-GAL4. The copulation duration was shortened by ~ 1.6 fold in the experimental line compared to the control (Control: Inactive TNT—Dsx^GAL4^, FLP^335^ > TNTin: n = 22; Silenced: Active TNT—Dsx^GAL4^,FLP^335^ > TNT: n = 24; black dots are individual values; red lines indicate the median with interquartile range; **p < 0.0029, ***p < 0.0001 by Mann Whitney test). (**H**) Pheromone requirement in the regulation of copulation duration. Mating pairs were set up between males expressing TNT in FLP^335^ restricted *dsx* neurons (Control—Inactive TNT and Silenced—Active TNT) and either females with ablated oenocytes (oe−) or their genetic controls (oe+). Black dots indicate individual values; red lines indicate the median with interquartile range. Overall difference in groups was observed (One-way ANOVA Kruskal–Wallis, p < 0.0001). When paired with control females (oe+), the copulation duration was shortened for the experimental line (active TNT) compared to the control line with an inactive TNT expressed in the subset of *dsx* neurons restricted by FLP^335^ (FLP^335^, dsx > TNTin: n = 33; FLP^335^, dsx > TNT: n = 40; posthoc Dunn’s multiple comparison test, p < 0.0001). When paired with oe− females, copulation duration was also shortened but less significantly in the experimental males compared to the control males (Control: Inactive TNT—Dsx^GAL4^, FLP^335^ > TNTin: n = 37; Silenced: Active TNT—Dsx^GAL4^, FLP^335^ > TNT: n = 28; posthoc Dunn’s multiple comparisons test, p = 0.0289). When paired with control males (Control: Dsx^GAL4^, FLP^335^ > TNTin), oe− females led to a small but significant reduction in copulation duration compared to the oe+ control (Dsx^GAL4^, FLP^335^ > TNTin vs oe+ : n = 33, Dsx^GAL4^, FLP^335^ > TNTin vs. oe−: n = 37; posthoc Dunn’s multiple comparisons test, p = 0.0088). When paired with experimental males (Silenced: Dsx^GAL4^, FLP^335^ > TNT), no significant difference was observed between oe+ and oe− females (Dsx^GAL4^, FLP^335^ > TNT vs oe+ : n = 40, Dsx^GAL4^, FLP^335^ > TNT vs. oe−: n = 28; posthoc Dunn’s multiple comparisons test, p = 0.9999).

To rule out the contributions of other neuronal populations to the copulation duration phenotype, we investigated other genetic combinations that target genitalia neurons more specifically. Previous research indicated that the genitalia neurons express *dsx*^11,12,40^. Indeed, we confirmed that the *fru*-MSN neurons are also *dsx* positive (Fig. 3A). Replacing *fru-*GAL4 with *dsx-*GAL4 in our genetic combination with FLP^335^ (Dsx^GAL4^, FLP^335^ > mCD8::GFP) eliminated all expression in the brain (Fig. 3C). In the VNC, Dsx^GAL4^, FLP^335^ > mCD8::GFP showed consistent expression only in the sexually dimorphic arbors originating from the foreleg GRNs and the genitalia neurons (Fig. 3B,D–F). Using this highly restrictive genetic combination, we silenced these neurons by TNT expression and evaluated the post-copulatory behaviors. Consistent with our hypothesis, copulation duration was still reduced at the same level for Dsx^GAL4^,FLP^335^ > TNT compared to the control (Fig. 3G). These results ruled out the involvement of CNS neurons in the copulation duration phenotype and indicate that that the *fru* neurons responsible for the phenotype are also *dsx* positive.

The characteristic male-specific midline crossing of the LAN1 arbors that originated from GRNs on the front legs is regulated by both *fru* and *dsx*^41^. These GRNs co-express the ion channel ppk25 that is critical for pheromone detection during early courtship steps^17^. To investigate whether the copulation duration phenotype requires female pheromone perception, we paired Dsx^GAL4^,FLP^335^ > TNT males with females whose pheromone-producing cells were ablated genetically via expression of the pro-apoptotic gene *hid* under an oenocyte-specific promoter^42^. Graphical evaluation of the four groups created by two levels of male (control:Inactive TNT/silenced:Active TNT) and two levels of female (control:oe+/no pheromones:oe−) suggests that there is an interaction between the effect of male (control/silenced neurons) and female (control/no pheromone) on copulation duration (Fig. 3H). Assumptions were not met for analysis with a two-way factorial ANOVA. An overall effect of the male factor can not be evaluated; in the presence of interaction the effect of male must be evaluated separately for each female group. Overall, a difference in copulation time in the four unique combinations was observed (one-way Kruskal Wallis ANOVA [nonparametric data (unequal variance and non-normal distribution)], p ≤ 0.0001). Experimental males with both GRNs and MSNs silenced (Active TNT) exhibited shortened copulation duration compared to the control (Inactive TNT) males, irrespective of whether they were paired with oenocyte-less females (oe−; Dunns’ test, p = 0.0289) or their genetic controls (oe+; Dunn’s test, p = 0.0088) (Fig. 3H). However, the copulation duration of the control males (TNT-in) is significantly shorter when paired with oe− females compared to control oe+ females (Fig. 3H). This suggests that female pheromone perception also contributes to the shortened copulation duration.

### Sensory information detected by peripheral senseory neurons is required for copulation persistence

**Figure 4:**
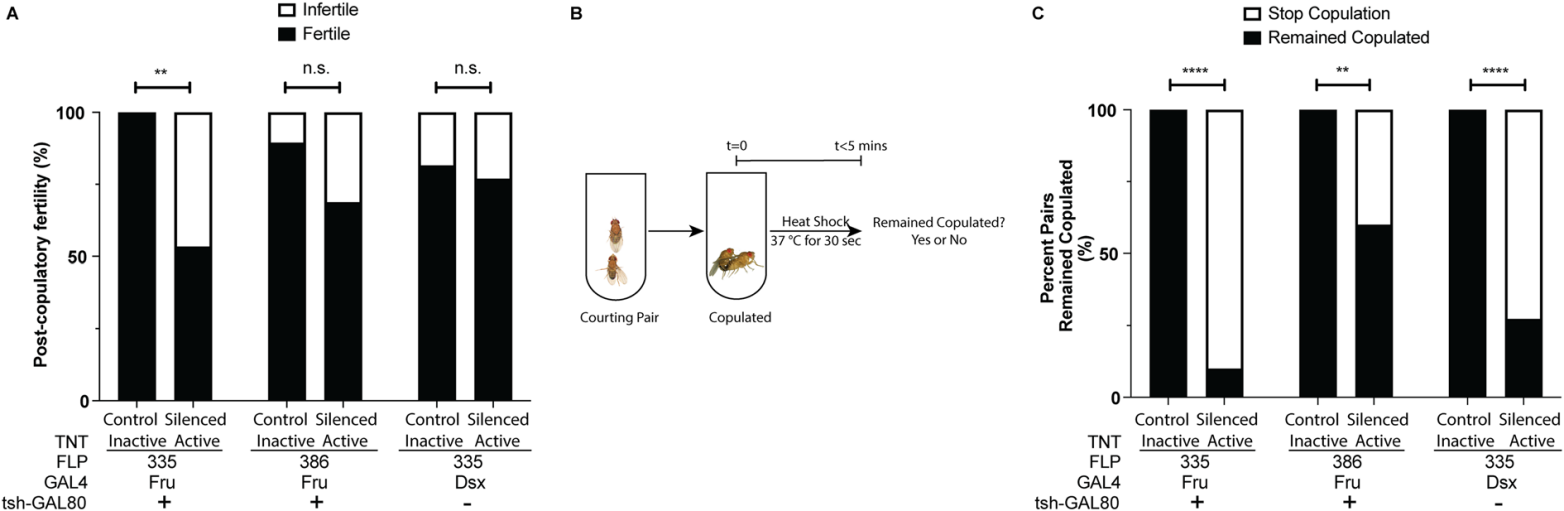
Sensory information detected by *fru*/*dsx*-MSNs is necessary to maintain copulation persistence. Mating pairs were set up between males expressing TNT in either FLP^335^ or FLP^386^ restricted *dsx* or *fru* neurons and CS females. (**A**) The fertility of the female after a single copulation event was assessed. Percentage of females that produced progeny (Fru^GAL4^, FLP^335^, tsh^GAL80^ > TNTin: n = 15; Fru^GAL4^, FLP^335^, tsh^GAL80^ > TNT: n = 15; Fru^GAL4^, FLP^386^, tsh^GAL80^ > TNTin: n = 19; Fru^GAL4^, FLP^386^, tsh^GAL80^ > TNT: n = 16; Dsx^GAL4^, FLP^335^ > TNTin: n = 27; Dsx^GAL4^, FLP^335^ > TNT: n = 26) n.s. = not significant, **p = 0.0063 by Fisher’s exact test. Females mated with males of Fru^GAL4^, FLP^335^, tsh^GAL80^ > TNT, which included both copulation duration regulating neural clusters (*fru*-sAbg-1 and *fru*/*dsx*-MSNs) were less likely to produce progeny compared to the TNT-in controls. On the other hand, females mated with males of Fru^GAL4^, FLP^386^, tsh^GAL80^ > TNT or Dsx^GAL4^, FLP^335^ > TNT, both of which include only one copulation duration regulating neural cluster (*fru*/*dsx*-MSNs) had normal fertility. (**B**–**C**) Copulation persistence was assessed by heat shocking copulating pairs within the first 5 min of copulation initiation. Percentage of mating pairs that remained copulated during heat shock (Fru^GAL4^, FLP^335^, tsh^GAL80^ > TNTin: n = 13; Fru^GAL4^, FLP^335^, tsh^GAL80^ > TNT: n = 10; Fru^GAL4^, FLP^386^, tsh^GAL80^ > TNTin: n = 18; Fru^GAL4^, FLP^386^, tsh^GAL80^ > TNT: n = 15; Dsx^GAL4^, FLP^335^ > TNTin: n = 30; Dsx^GAL4^, FLP^335^ > TNT: n = 22) ****p < 0.0001, **p = 0.0045, by Fisher’s exact test. TNT-silencing of neurons by the three genetic combinations all resulted in higher percentage of males that terminated copulation upon heat shock compared to their respective TNT-in controls.

Next, we asked the biological relevance of a shortened copulation duration as a result of silencing *fru/dsx* GRNs and MSNs. In a productive copulation pairing, duration must be long enough for the transfer of sperm and accessory gland fluid. In the three genetic combinations presented here, the median copulation duration for the experimental males is 10–14 min, which is longer than the minimal time (~ 8 min) necessary for sperm transfer^26,27^. To evaluate if sperm is successfully transferred from the test male to the target female, we quantified the number of copulation pairings that resulted in fertilization after a single copulation event between a test male and a virgin Canton-S female. Compared to inactive TNT controls, a significantly lower percentage of males where both copulation duration regulating neural clusters (sAbg-1 and MSNs) are silenced (Fru^GAL4^, FLP^335^, tsh^GAL80^ > TNT males) could fertilize the virgin females. In contrast, males in which sAbg-1 are not manipulated but MSNs are silenced (Fru^GAL4^, FLP^386^, tsh^GAL80^ > TNT and Dsx^GAL4^, FLP^335^ > TNT males) have relatively normal levels of fertilization rates compared to their respective inactive TNT controls (Fig. 4A). Therefore, post-copulatory fertility is unaffected by the shortened copulation duration due to the silencing of *fru/dsx* GRNs and MSNs.

If fertility is unaffected, we wondered if peak copulation persistence is still possible without sensory input from GRNs and MSNs. To evaluate if copulation persistence is affected by the silencing of the *fru*/*dsx* GRNs and MSNs, we applied heat shock as the stress stimulus to copulating flies when copulation persistence is at its peak. We heat-shocked pairs of flies 5 min after the onset of copulation and quantified the number of pairs that disengaged (Fig. 4B). While a large number of pairs in which the *fru*/*dsx* GRNs and MSNs were silenced by our three genetic combinations (Fru^GAL4^, FLP^335^, tsh^GAL80^ > TNT; Fru^GAL4^, FLP^386^,tsh^GAL80^ > TNT; Dsx^GAL4^, FLP^335^ > TNT) terminated copulation, all pairs with the corresponding control males remained copulating (Fig. 4C). These results indicate that the *fru*/*dsx* GRNs and MSNs are important in maintaining copulation persistence in response to environmental stress before sperm transfer is complete.

## Discussion

Different neuronal populations have been identified to control three distinct aspects of male copulatory behaviors: genital coupling, copulation duration, and sperm transfer^27,28,40^. Here, we have characterized the *fru*/*dsx-*MSNs at the genitalia. We also identified novel function of the peripheral sensory neurons at the front legs (GRNs) and genitalia (MSNs) in regulating copulation duration and maintaining copulation persistence in the presence of environmental stressors.

**Figure 5:**
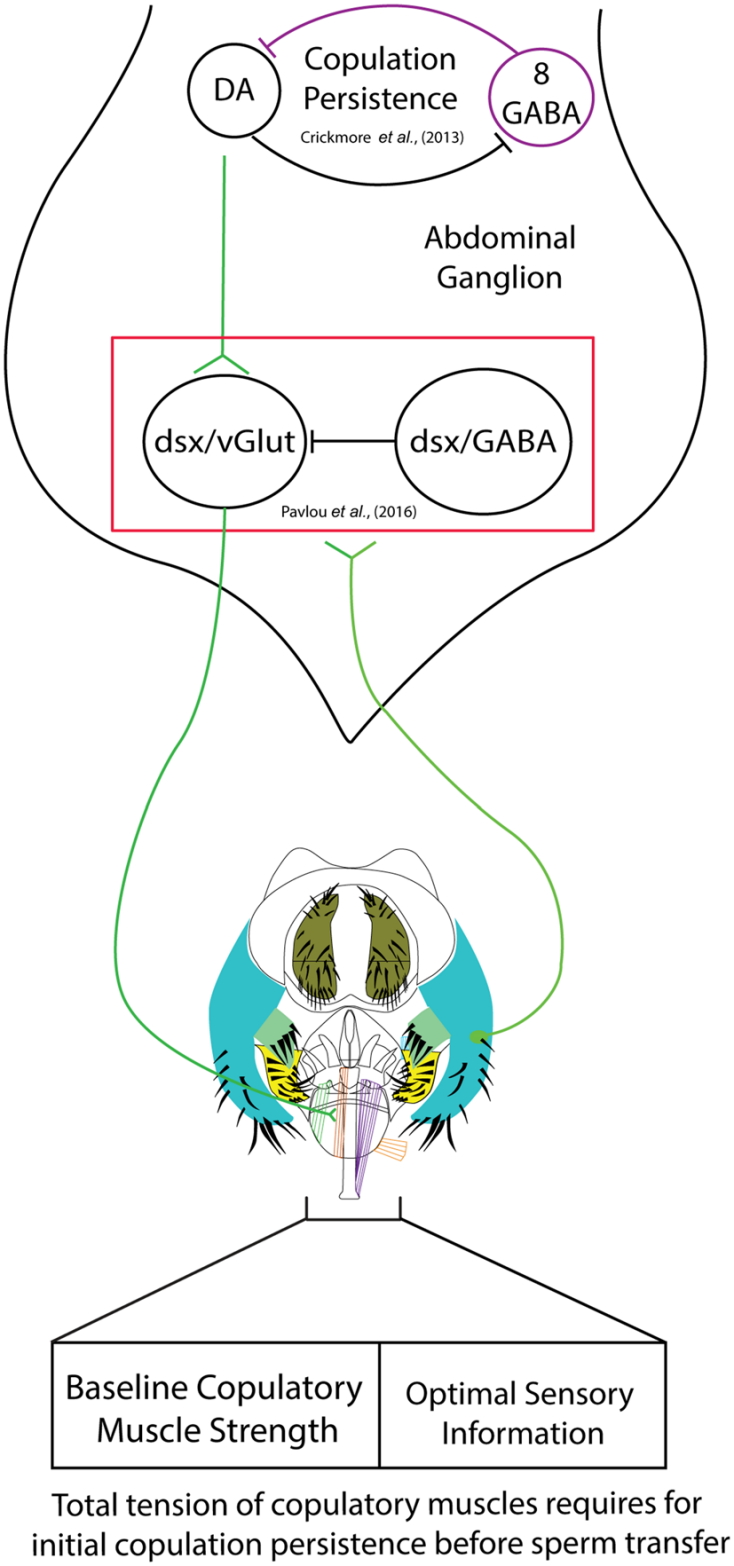
Proposed model of how sensory information from the MSNs is incorporated into the neural circuit that governs copulation persistence. Genital coupling requires the activation of the *dsx*/vGlut motor neurons that innervate the copulatory muscles. The resulting muscular tension is proportional to the activity level of the dsx/vGlut motor neurons, which are antagonistically regulated by *dsx*/GABA interneurons. These copulation regulating neurons are functionally connected to the axonal terminals of sensory neurons from the genitalia bristles^40^. We propose that a suboptimal sensory input will reduce the total muscular tension due to less activation of the *dsx*/vGlut motor neurons or more activation of the *dsx*/GABA interneurons. Copulation persistence ensures the maintenance of copulation before sperm transfer in the presence of stressful stimulus and is regulated by opposing actions of 8 *dsx*/GABA and the dopaminergic system in the ventral cord^24^. This persistence requires optimal sensory input from the genitalia bristles.

Retrograde labeling of various genitalia MSNs have shown that most of these neurons project only to the abdominal ganglion except for one neuron from the surstylus (clasper) that projects all the way to the suboesophageal region of the brain^40^. Since we did not observe any arborizations in the brain, the copulation phenotype appears to be regulated by a subset of the *fru*/*dsx*^−^ MSNs at the surstylus (claspers) and epandrial ventral lobe (lateral plate) that project specifically to the abdominal ganglion. In addition, the retrograde labeling experiment showed that the axonal terminals of the genitalia neurons juxtapose the dendrites of the abdominal *dsx* glutamatergic (dsx/vGlut-Abg) and GABAergic (dsx/GABA-Abg) neurons^40^. Moreover, artificial mechanical stimulation of the genitalia with a minuten pin activated both dsx/vGlut-Abg and dsx/GABA-Abg neurons^40^. Therefore, we can infer that *fru*/*dsx*-MSNs neurons make functional synaptic connections to both dsx/vGlut and dsx/GABA neurons in the abdominal ganglion (Fig. 5). The shortened copulation duration phenotype when *fru*/*dsx-*MSNs are silenced is a result of dsx/vGlut-Abg and/or dsx/GABA-Abg not receiving sensory feedback signals from the genitalia.

dsx/vGlut-Abg are all motor neurons that innervate the phallic and periphallic musculature responsible for genital attachment^40^. On the other hand, dsx/GABA-Abg is comprised of a heterogeneous population of interneurons with different functions. While some of these interneurons inhibit the activity of dsx/vGlut-Abg to terminate copulation^40^, others have different functional roles, such as regulating copulation persistence^24^. Therefore, sensory signals received from the *fru/dsx*-MSNs can influence the relative activity level of dsx/vGlut-Abg and dsx/GABA-Abg neurons with opposing actions and modulate the overall tension of the copulatory muscles. In the absence of sensory information, the baseline muscle strength is sufficient to maintain genital coupling long enough for sperm transfer since males with *fru*/*dsx*-MSNs silenced has normal fertility (Fig. 4A). However, our results show that the sensory information encoding the male’s correct engagement in copulation is necessary to achieve peak copulation persistence before sperm is transferred (Figs. 4B, 5). Sensory information—provided by fru/dsx-MSNs—might be a way to measure the quality of the copulation. Genital coupling will lead to bending of the bristle hairs and activate the *fru*/*dsx*-MSNs. Which bristle hair gets stimulated and the strength of the stimulation will depend on whether genital coupling is established and the morphology of the female genitalia. For example, an abnormal amount of pressure received by a bristle hair could send less signal to activate the copulatory muscle innervated by dsx-vGlut-Abg. Similarly, a wrong set of bristles bent could activate the dsx-GABA-Abg neurons that inhibit the dsx-vGlut-Abg. A suboptimal activation decreases the total muscular tension during genital coupling. Copulation persistence is a result of the fly’s ability to analyze the tradeoff of maintaining copulation during exposure to an environmental stressor. Sensory information that signals the quality of the copulation provides a critical factor in this assessment.

Our results showing males’ perception of female pheromone contributes to the regulation of copulation time is surprising because our previous work showed that genetically silencing olfactory receptor or gustatory receptor independently did not result in shortening of copulation duration^28^. Since females defective in pheromone synthesis would lack both volatile (detected by olfactory receptor) and non-volatile (detected by gustatory receptor), it is possible that males use olfactory or gustatory cues to evaluate female fitness. In the absence of both sensory cues, the males assess that the female has poor fitness for reproductive success and decide to shorten the copulation duration in order to select for an alternative mate. Non-volatile pheromones are cuticular hydrocarbons (CHCs) synthesized from long-chain fatty acids. Therefore, abnormal distribution of CHCs could reflect metabolic deficiency. In line with this explanation, diet has been shown to influence copulation duration by altering the gut microbiome, whose metabolism changes the cuticular hydrocarbon profiles that could affect the amount of sex pheromones present^30,31^.

As is observed in many species, copulation duration reflects the reproductive investment of the males^33^. Although sperm transfer occurs in the first 5 min of copulation^24,25^, mating lasts much longer to an average of 20 min in *Drosophila melanogaster*^22^*.* Males can adjust their mating time in response to their social-sexual environment. Males exposed to other males prior to mating copulate longer compared to socially isolated males^32^. The prolonged mating time resulted in females that laid more eggs and reduced the likelihood of remating^32^. Prolonged mating time is necessary for the transfer of seminal proteins that ensures sperm competitiveness of the mating male against other rivals, particularly for heterospecific males that can cause reproductive interference^43^. Synthesis of seminal fluid is energetically costly to the male and therefore, the allocation of seminal fluid must be strategized. Sustaining copulation with a suboptimal mate is a loss of opportunity to gain a better mate. The evaluation of an optimal copulation time requires a neural circuit that processes sensory information from the environment and triggers the motor program for copulation. Males require at least two sensory cues from sound, smell, or touch to recognize the presence of rivals and respond by increasing mating time^44^. With our genetic tools to specifically silence the front leg fru-dsx-gustatory neurons and genitalia mechanosensory neurons, it will be interesting to test whether these males will lose the ability to perceive the presence of rivals and respond with a lengthened copulation duration.

Our work revealed the importance of sensory information collected by both front leg dsx/fru GRNs and genitalia MSNs in regulating copulation duration. To further dissect the functional role of gustation and mechanosensation, new genetic strategies to target gene expression to either GRNs and MSNs is necessary. The recently published transcriptomic atlas for both the ventral nerve cord^45^ and the male genitalia^46^ provides new possibilities to develop new tools that will allow further investigation of the neural mechanism of how sensory information is encoded and processed in the CNS.

## Materials and methods

### Fly strains

The following strains were used in this study: *fru-GAL4*^7^, *dsx-GAL4*^11^*, UAS* > *stop* > *TNTin*, *UAS* > *stop* > *TNT*^7^, *UAS* > *stop* > *mCD8::GFP*^9^, *FLP*^*335*^ and *FLP*^*386*^ were generated as described in previous study^28^, *cha-GAL80*^47^, *tsh-GAL80* from Julie Simpson, and Canton-S strain from the Bloomington Stock Center, Bloomington, IN. Oenocyte less (oe−) flies and their controls were generated as previously described^42^.

### Immunohistochemistry

Dissection of 3–7 day old adult flies and immunohistochemistry of the adult nervous system were carried out as described previously^48^ with some modifications in primary and secondary antibodies. Dissection of male reproductive organs was performed on Sylgard plate covered with 1 × PBS. To obtain male genitalia, the lower half of the abdomen were dissected and fixed in 4% PFA at room temperature for 20–30 min. After which the PFA was replaced with 1xPBS. The genitalia were cut out with a pair of microscissors to ensure a flat surface for mounting. Dissected samples were fixed and proceeded with immunostaining as described previously^48^. The following primary and secondary antibodies were used in this study: rat polyclonal anti-mCD8 (1:100; Caltag, Burlingame, CA), mouse anti-GFP (1:500, Life technologies), mouse anti-nc82 (1:20; Hybridoma Bank)^49^, rabbit anti-Dsx^M^ (1:2000) (kindly supplied by Brian Oliver laboratory, NIH), anti-rat IgG conjugated with Alexa Fluor 488 (1:300; Invitrogen), anti-mouse IgG conjugated with Alexa Fluor 488 (1:300 Invitrogen), and anti-rabbit IgG conjugated with Alexa Fluor 594 (1:300; Invitrogen).

### Microscopy

Images of the ventral cord, the reproductive tissues, and genitalia were acquired using an Olympus Fluoview FV1000 confocal microscope with a 20X objective. ImageJ was used to stitch the overlapping images together. All other images were acquired using a Zeiss LSM 700 confocal microscope. For the genitalia, lambda stacks from 490 to 600 nm at 20 nm intervals were acquired. The autofluorescence signals were unmixed from the 488 nm signals using the linear unmixing algorithm in the Zen Black software.

### Behavioral assays

#### Husbandry

Flies were raised on standard cornmeal medium and kept on a 12 h:12 h day:night cycle at 25 °C in ambient relative humidity. Each newly eclosed adult was collected and aged for 3–7 days in an isolation vial (16 × 100-mm) supplied with ~ 2 ml of fly food. Virgin, wildtype Canton-S females were aged in groups of 20–40 for 3–7 days. All behavioral experiments were performed at 25 °C with ~ 50% humidity during the first 3 h. after lights on.

#### Copulation assay

Copulation assays were performed in 12-well plates (Thermo Scientific BioLite Multidish). A square glass plate covering four wells was used as the lid. Assays were performed in 4 wells with ~ 5 ml standard fly food to maintain humidity in each well. An experimental male was paired with a virgin female and the courtship behavior was videotaped for at least an hour. If a pair did not copulate within an hour, it was considered unsuccessful. Copulation duration was calculated from the beginning of genital coupling until the male was dismounted from the female.

#### Fertility assay

Freshly hatched males were isolated into a glass tube (16 × 100 mm) with 2 ml of food and kept for 4–5 days at standard conditions (25 °C, 50% RH). A 6–7 day old CS virgin female was introduced into each tube using an aspirator. After transferring the vials back to standard conditions, the flies were allowed to interact for 1 h and observed every 10 min for successful copulation. Tubes with pairs that did not copulate were discarded. For the rest of the tubes, males were removed by quick anaesthetization and females were allowed to lay eggs for 24 h and then discarded. Fertility was recorded after 7 days by observing the presence of progeny.

#### Persistence assay

A single experimental male was paired with a wildtype CS virgin female in a glass tube (16 × 100 mm) with or without food. If copulated in a vial with food, the pairs were transferred to an empty vial before heat shock to ensure efficient heat transfer. Each pair that successfully copulated (within 5 min) was subjected to heat stress by submersing the empty glass vial (16 × 100 mm) in a 37 °C water bath for 30 s. Frequency of copulation termination was recorded.

### Statistics analysis

Prism version 9.3.1 (https://www.graphpad.com/scientific-software/prism/) was used for all statistical analysis. For Fig. 3H, presence of interaction between factors was assessed visually (data from groups at different levels of one variable showed differing observed mean values and distributions for separate levels of another variable). Results from the two way factorial design of this study (control vs. silenced males x control vs. pheromones-less females) were assessed for fit to a two way factorial ANOVA (normality and homogeneous variance of residuals). For nonparametric data, a one way Kruskal–Wallis ANOVA (nonparametric) was fit to combinations created of levels for each of the two factors (control vs. silenced males x control vs pheromone-less females) when interaction was present. Post hoc pairwise comparisons, controlling for multiple comparisons, were performed using Dunn’s test for multiple comparisons.

## Supplementary Information

Supplementary Video 1.
